# The effects of boar on susceptibility to swine inflammation and necrosis syndrome in piglets

**DOI:** 10.1186/s40813-021-00194-2

**Published:** 2021-01-28

**Authors:** Josef Kuehling, Kathrin Eisenhofer, Mirjam Lechner, Sabrina Becker, Hermann Willems, Gerald Reiner

**Affiliations:** 1 Department of Veterinary Clinical Sciences, Clinic for Swine, Justus-Liebig-University, Frankfurter Strasse 112, 35392 Giessen, Germany; 2 UEG Hohenlohe, Am Wasen 20, 91567 Herrieden, Germany

**Keywords:** Inflammation and necrosis syndrome, Animal welfare, Boar, Swine

## Abstract

Inflammation and necrosis can appear in pigs in several parts of the body simultaneously. The signs can affect newborns, suckling piglets and older pigs, and recent studies suggest that the syndrome is primarily endogenous. Inflammation and necrosis indicate impaired animal welfare, and thus should be controlled in pig production. This can be achieved by improving husbandry conditions. However, the variation in signs also appears to have a genetic component. The aim of the present study was therefore to test the effects of different boars from the Duroc and Pietrain breeds on the prevalence of swine inflammation and necrosis syndrome in their offspring. For this purpose, 646 suckling pigs from 39 sows (two herds) and 19 boars were made available. On the third day of life, the piglets were examined for clinical signs of inflammation and necrosis at tail base, tail tip, ears, face, teats, navel and claws. For the evaluation, we included the boar within the breed and the breed as fixed effects and the sow within the herd as random effects. More than 70% of the piglets were affected at the tail base, ears, coronary bands and heels. Bristle loss, swelling, redness, venous congestion and claw wall bleeding occurred most frequently. Exudation and necrosis affected fewer piglets. None of the piglets was completely free from signs of SINS. Offspring from Duroc boars had significantly lower SINS scores (4.87 ± 0.44) than offspring from Pietrain boars (10.13 ± 0.12). Within the Pietrain breed, significant effects of the boar were observed on inflammation and necrosis levels. Under the present study conditions, using Duroc boars instead of Pietrain boars resulted in a 59% reduction in the SINS scores of their offspring. The SINS score in the offspring of the most favourable Pietrain boar was almost 40% lower than that of offspring in the least favourable. These findings confirm considerable genetic effects on the outcome of SINS under a given husbandry. Further studies are necessary to characterise the genetic effects in detail and to make them useful to combat the syndrome.

## Background

Signs of inflammation and loss of tail integrity indicate serious impairment of animal welfare [1, 2], and preserving animal welfare is one of the major challenges facing modern pig farming. Tail biting is a very prevalent undesirable behaviour that has been identified as a major source of significant reduction in tail integrity, especially in growing pigs [3–7]. Even with intensive use of available measures, 25 to 70% of animals may have damaged tails (e.g., [8–10]). However, the term ‘tail-biting’ covers a mixture of different types and drives [3]. Additionally, evidence from research and practice suggests that tail lesions might not only be caused by tail biting, but also by inflammation and necrosis, which can occur without any action by other pigs [11–17]. These lesions are also not limited to the tail, but can be observed in ears, heels and soles, claw coronary bands, teats, navel, vulva and face. Due to the syndrome-like combination of different body parts and the clinical domination of inflammation and necrosis in these areas, this clinical outcome has been coined swine inflammation and necrosis syndrome (SINS [14]). Inflammation has been histologically proven as the cause of SINS [16, 17]. The clinical and histological evidence of SINS in newborn piglets [16] shows that the symptomatology can develop without biting or technopathies such as unfavourable floor conditions, even if such effects can play a decisive role in the final expression of the signs [18].

The grade of SINS can be affected by husbandry conditions and by the quality of the sow [17]. Practical experience suggests that there might also be genetic effects on the expression of SINS, and effects of sow genetics have already been proven [14]. The aim of the present study was to investigate the influence of the boar breed and the individual boar on the manifestation of SINS in the offspring from a uniform sow basis.

## Materials and methods

### Experiment and experimental animals

The animal experiment was carried out in the conventional pig breeding stables of the Oberer Hardthof teaching and research station at Justus-Liebig University Giessen, Germany and at a closed herd farm in Lower Saxony under the approval of the authorities in Giessen, Germany with file numbers V54–19 c 20 15 h 02 Gi 18/15 kTV 3/2019 and V 54–19 c 20 15 h 02 GI 18/15 kTV 4/2020.

A total of 646 piglets from 39 sows and 19 boars were available for this study. The piglets were from two herds (herd 1: *n* = 245; herd 2: *n* = 401). They were examined in their third day of life. Both herds had no history of exudative epidermitis before or after the present study. The sows for herd 1 were from a uniform Topigs x German Landrace genetic. The sows of herd 2 were DAN-Bred. All sows were artificially inseminated.

### Herd1

In the breeding centre, the sows were fixed in stands on concrete slatted floor until the 28th day of pregnancy. They were fed liquid food in a longitudinal trough via a Spotmix feeder. Water was available via Aqua Level drinking troughs.

In the waiting position, the sows stood in a 145 m^2^ compartment on concrete slatted floor with separate lying areas. Feed could be requested by feeding on demand. Water supply was ensured via nipple and Aqua Level drinking troughs.

The sows were vaccinated against erysipelas and parvovirus on the 14th day of lactation (Porcilis Ery + Parvo, MSD, Germany). A vaccination against *Clostridium perfringens* was performed two weeks before birth (Clostriporc A, IDT, Germany). The SPF herd was free from porcine reproductive and respiratory syndrome virus (PRRSV), *Actinobacillus pleuropneumoniae*, *Lawsonia intracellularis, Brachyspira hyodysenteriae* and *Brachyspira pilosicoli.*

In the farrowing house, sows and suckling piglets were kept in 4.8 m^2^ farrowing pens with a plastic slatted floor. The sows were fixed in a farrowing crate with a flat surface. The sows’ floor was a slatted cast iron floor. Feed was offered via a Spotmix feeder in the trough. Nipple drinkers and mother-child basin drinkers provided a water supply for the animals.

#### Feeding

The composition of the gestation feed was 12.5% crude protein, 2.8% crude fat, 7.0% crude fibre, 4.4% raw ash, 0.66% calcium, 0.46% phosphorus, 0.15% sodium, 0.7% lysine, 0.18% methionine and an energy content of 12,02% ME MJ/kg. The Ingredients of the lactation diet were 16.0% crude protein, 3.3% crude fat, 5.0% crude fibre, 6.71% raw ash, 0.79% calcium, 0.54% phosphorus, 0.21% sodium, 0.94% lysine. 0.3% methionine and an energy content of 12.71 MJ ME/kg.

### Herd 2

The breeding centre was built with a concrete slatted floor. The sows were fixed in metal stands till the 28th day of gestation. Feeding was performed with a longitudinal through in front of the fixation stands. The feed amount for every individual sow was allocated with a volume doser. Water was available via Aqua Level drinking troughs. The waiting position was a 60 m^2^ sized room with particular concrete slatted floor. Self-catching feeding bays were installed on two opposites walls of the stable. In front of the bays a longitudinal trough was positioned for sow feeding. The feed dropped down into the trough through a volume doser. Water supply was ensured by Aqua Level drinking troughs.

The sows were vaccinated against erysipelas and parvovirus on the 14th day of lactation (Porcilis Ery + Parvo, MSD, Germany). Sows were vaccinated against PRRS (Porcilis PRRS, MSD, Germany) and Influenza A (Respiporc Flu 3, IDT, Germany) every 3rd month. In the farrowing house, sows and suckling piglets were kept in 5 m^2^ farrowing pens with a plastic slatted floor (slat width 11 mm). The sows were fixed in a farrowing crate with a flat surface. The sows’ floor was a slatted cast iron floor. The feed was offered via a volume doser to the trough. Nipple drinkers and mother-child basin drinkers ensured the water supply for the animals.

#### Feeding

The sows were fed with a commercial gestation and lactation diet from a local mill in Lower Saxony. The ingredients were orientated for the Recommendations of the German Agricultural Association (DLG). The composition of the gestation feed was 14.0% crude protein, 3.0% crude fat, 7.0% crude fibre, 6.5% raw ash, 0.7% calcium, 0.45% phosphorus, 0.25% sodium, 0.78% lysine, 0.28% methionine and an energy content of 12,2% ME MJ/kg. The ingredients of the lactation diet were 16.5% crude protein, 4.5% crude fat, 5.5% crude fibre, 6.3% raw ash, 0.8% calcium, 0.55% phosphorus, 0.25% sodium, 0.96% lysine. 0.31% methionine and an energy content of 13.0 MJ ME/kg.

#### Boars

The study aimed to compare extreme boars in terms of the susceptibility of their offspring to SINS. The aim was not to generate representative data for boar lines of breeding companies. Thus, boars were specifically selected which, on the basis of field observations over the last four years, seemed to have particularly low or particularly high SINS levels in their offspring compared to other boars. In this way, 19 boars of two breeds (4 Duroc boars and 15 Pietrain boars) were used on 39 sows of two herds. The boars came from 7 different, international breeding companies. Detailed information on the breeding companies is not provided because no representative result for the breeding companies can be derived from the targeted selection of boars. The boars were used in both sow herds and had an average of 34 offspring with 4 sows. The sows were artificially inseminated. In order to increase the sow-boar combinations on the basis of a manageable number of piglets, all boars were used in pairs as mixed semen. This means that piglets from two different boars were present in each litter at the same time. Of course, not all possible boar combinations could be created, but the number of sow-boar combinations was doubled. All piglets were assigned to the correct boar by paternity testing.

#### Paternity testing

Paternity testing was based on the genetic matches between offspring and boars. The piglets were tail-docked one day after clinical scoring. DNA was extracted as described in Reiner et al. [19] from the docked tail tissue. Genotyping was done with 14 microsatellites in 2 multiplex PCRs and microsatellite alleles were determined by capillary gel electrophoresis [19].

#### Clinical scoring

Inflammation and necrosis were clinically assessed as described by Reiner et al. [14]. The piglets were scored on the 3rd day of life to ensure comparability with other studies and because clinical signs were clearly visible during this period in all previous studies, but the piglets were not yet exposed to environmental effects as weaners and fatteners. For time reasons and to minimize the animal load, clinical signs were recorded using a digital camera (Canon EOS DC 8.1 V, Canon) according to a standardized scheme for later detailed evaluation of the images (Windows Media Player, Version 12, Microsoft GmbH, Germany).

Clinical alterations in the tail base and tail tip, the ears, the teats and navel, coronary bands, wall horn, ball and sole of the feet as well as the face were assessed individually. However, the scoring was more detailed than in Kühling et al. [16]. The following clinical characteristics were considered and scored 0, if the sign was not visible or 1, if the sign was visible. The tail was scored for swelling, redness, rhagades, exudation, bleeding, tail necrosis and ring-shaped constrictions. The tail base was separately screened and scored for the presence of bristles, swelling of the tail base, redness of the tail base, exudation and clinical signs of necrosis. Ears were scored for the presence of bristles, congested ear veins and necrosis of the ears. Teats were scored for scab formation, swelling, reddening, necrosis and congested blood vessels. The navel was scored for redness and swelling. The face was scored for oedema around the eyes and nasal edema. Each claw was individually scored for wall bulging, wall bleeding, reddening of the heel, heel bleeding and inflammation of the coronary band. All scores were assigned by two experienced persons together. Inter and intra-observer effects were not estimated.

**Fig. 1:**
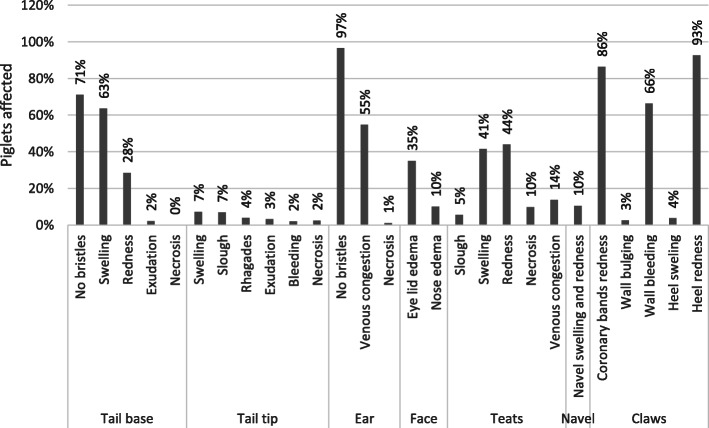
Prevalence of inflammation and necrosis in different body parts of the three-day-old piglets

The examined binary scores were presented by organ system as percentage of affected piglets. In addition, the percentages of piglets with the respective findings within an organ system were summarised as stacked bar charts (summed up percentage of affected piglets) to show the effects of the breed. All recorded binary scores (see Fig. 1) were summed up unweighted to the SINS score. This resulted in possible SINS scores between 0 and 27 for each piglet.

**Table 1:** Significant differences in the SINS outcome between boars after Bonferroni correction

Boar number	Different from boar numbers (P < 0.05)
1	4–7, 19
2	3, 4–7, 16, 19
3	2, 4–6, 8, 15, 19
4	1–3, 8–18, 20
5	1–3, 8, 9, 11, 12, 15–18, 20
6	1–3, 8, 9, 11, 12, 15, 18, 20
7	1, 2, 8, 15, 17, 18, 20
8	4–7, 16, 19
9	4–6
10	4
11	4, 5
12	4–6
13	4
14	4
15	3, 4–7, 16, 19
16	2, 4, 5, 8, 15, 19
17	4–7, 19
18	4–7, 19
19	1–3, 8, 15, 17–20
20	4–7, 19

#### Statistics

Data were analysed using IBM-SPSS, Version 27 (IBM, Munich, Germany). All composed organ scores were checked using QQ-plots, skewness and kurtosis. The residues of all variables were found to be largely normally distributed. Scores were analysed using a mixed-effect linear model with the boar within breed and the breed as fixed effects and the sow within herd and the herd as random effects. Results were presented as least square means with standard errors. Binary data were calculated with a generalised mixed model considering the effects as in the linear model. All data were Bonferroni corrected.

## Results

SINS was scored in 646 piglets from 39 sows and 19 boars from 7 breeding companies. Over 70% of the piglets showed affections at the tail base, ears, coronary bands and heels (Fig. 1). In over 40% of the animals the teats were affected. Only the tail tip and the navel showed alterations in less than 10 % of the animals. The most common signs were a lack of bristles, swelling, redness and bleeding into the claw wall. Severe alterations such as rhagades, exudation or necrosis occurred only in individual piglets.

**Fig. 2:**
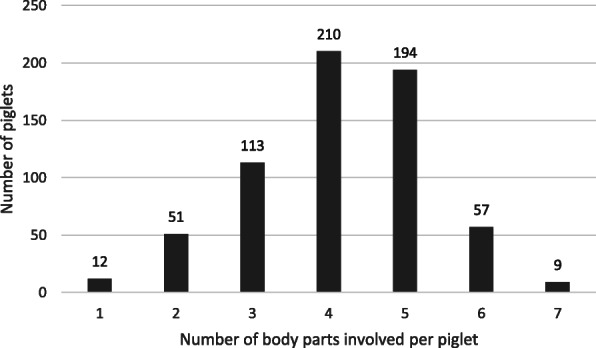
Number of piglets and number of body parts simultaneously involved

None of the 646 piglets was completely free from signs. Of seven body parts examined (tail base, tail tip, face, ear, teats, navel and claws), on average the piglets were affected in 3.8 ± 1.07 (mean ± SD) body parts simultaneously. Forty percent of the piglets were affected in at least 5 of 7 body parts (Fig. 2).

**Fig. 3:**
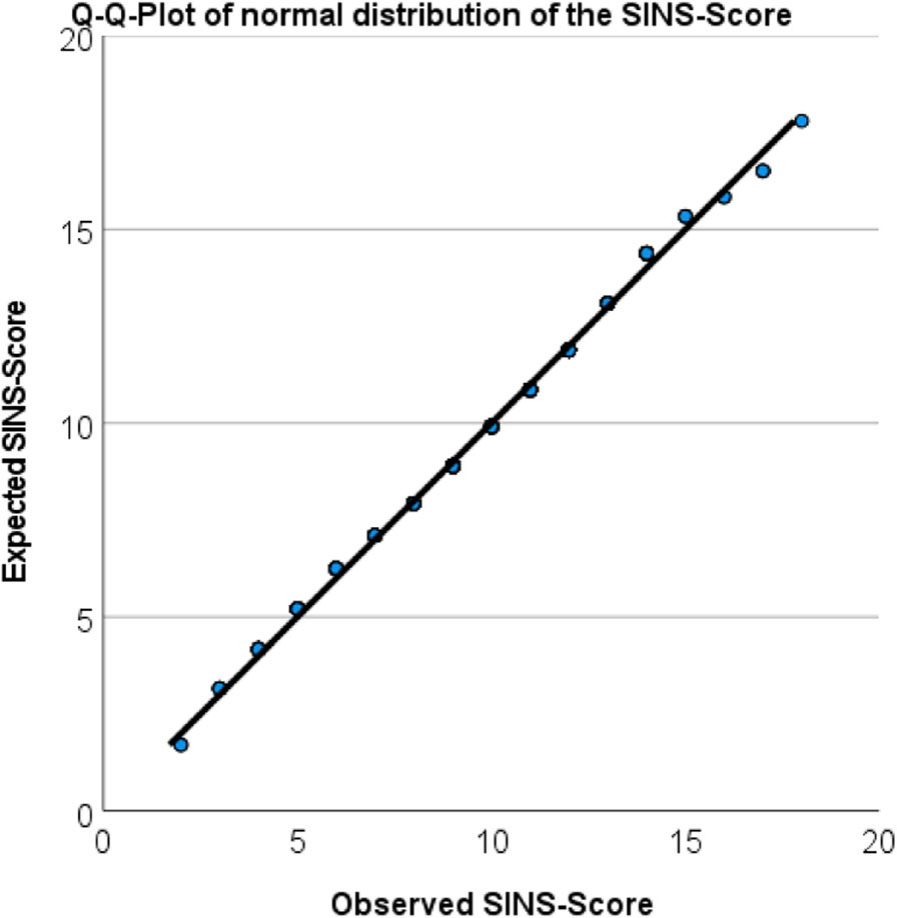
Q-Q plot of the normal distribution of the SINS (swine inflammation and necrosis syndrome) score

**Fig. 4:**
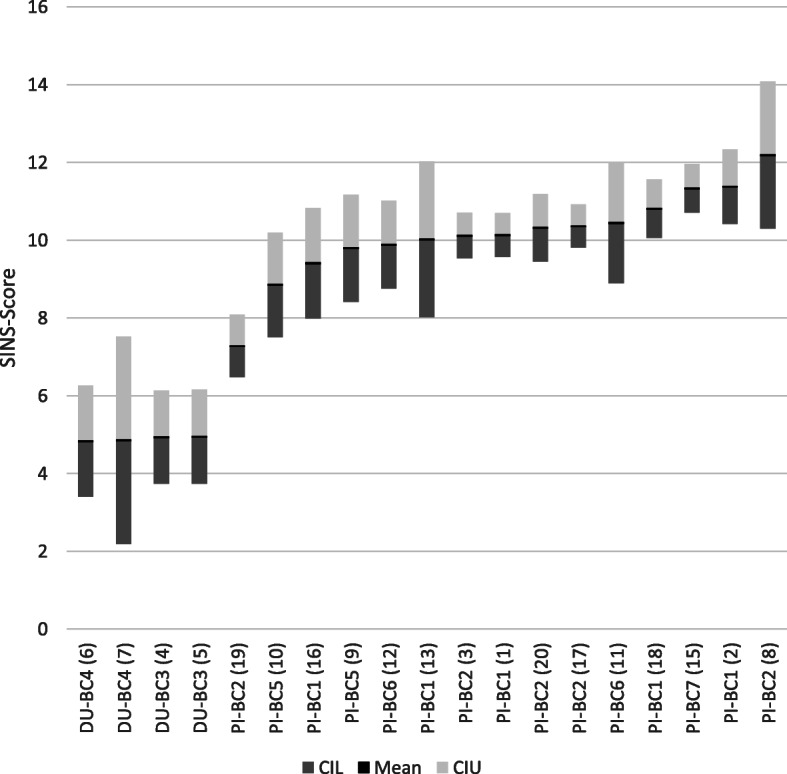
SINS (swine inflammation and necrosis syndrome) score of the offspring of individual boars by breed and breeding company. DU: Duroc, PI: Pietrain; BC: breeding company (number); boar number in brackets. The figure shows means with lower (CIL) and upper (CIU) confidence intervals

The SINS score was normally distributed (Fig. 3) with a mean of 9.5, a standard deviation of 2.8, a minimum of 2 and a maximum of 18 (data not shown). The SINS score was significantly affected by the boars' breed (*p* < 0.001), the breeding company (*p* < 0.001) and the boar (*p* < 0.001) (Fig. 4, Table 1). Offspring from Duroc boars (boars 4–7) had significantly lower SINS scores than offspring from Pietrain boars. Piglets from different Duroc boars were not differently affected, although SINS scores varied between 2.2 and 7.5 in one of the boars. Within Pietrain boars, the SINS scores of their offspring varied substantially. Offspring from boars 8, 2 and 15 differed significantly from those of boars 19 and 16 (Table 1). Piglets from the other eight Pietrain boars, with mean SINS scores around 10, did not differ significantly within breed. Offspring from boars of different breeding companies did not differ significantly within both breeds (data not shown). Offspring from Duroc boars had SINS scores of 4.87 ± 0.44 (mean ± SE) which was significantly less than the values of piglets from Pietrain boars (10.13 ± 0.12).

**Fig. 5:**
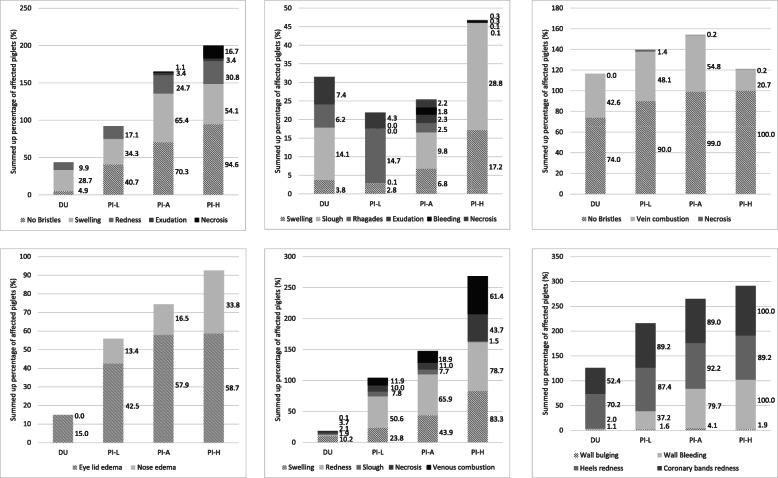
Cumulative percentage of three-day-old suckling piglets with clinical signs of inflammation and necrosis. **a** At the tail base. Percentages for the loss of bristles, swelling, redness, exudation and necrosis are given beside the stacked columns. DU: average for offspring of four Duroc boars. PI: offspring of Pietrain boars; L: offspring with least SINS signs (offspring from one boar, number 25); A: average of all Pietrain offspring; H: offspring with highest grades of SINS signs (one boar, number 8). **b** At the tail tip (complete tail without tail base). Percentages of piglets with swelling, slough, rhagades, exudation, bleeding and necrosis are shown beside the stacked columns. DU: average for offspring of four Duroc boars. PI: offspring of Pietrain boars; L: offspring with least SINS signs (offspring from one boar, number 25); A: average of all Pietrain offspring; H: offspring with highest grades of SINS signs (one boar, number 8). **c** At the ears. Percentages of piglets with lost bristles, vein combustions and necrosis are shown beside the stacked columns. DU: average for offspring of four Duroc boars. PI: offspring of Pietrain boars; L: offspring with least SINS signs (offspring from one boar, number 25); A: average of all Pietrain offspring; H: offspring with highest grades of SINS signs (one boar, number 8). **d** At the face. Percentages of piglets with eyelid oedema and nose oedema are shown beside the stacked columns. DU: average for offspring of four Duroc boars. PI: offspring of Pietrain boars; L: offspring with least SINS signs (offspring from one boar, number 25); A: average of all Pietrain offspring; H: offspring with highest grades of SINS signs (one boar, number 8). **e** At the teats. Percentages of piglets with swelling, redness, slough, necrosis and venous combustions are shown beside the stacked columns. DU: average for offspring of four Duroc boars. PI: offspring of Pietrain boars; L: offspring with least SINS signs (offspring from one boar, number 25); A: average of all Pietrain offspring; H: offspring with highest grades of SINS signs (one boar, number 8). **f **At the claws. Percentages of piglets with wall bulging, wall bleeding, redness of heels and coronary bands are shown beside the stacked columns. DU: average for offspring of four Duroc boars. PI: offspring of Pietrain boars; L: offspring with least SINS signs (offspring from one boar, number 25); A: average of all Pietrain offspring; H: offspring with highest grades of SINS signs (one boar, number 8)

It was not just the total SINS scores which were found to be significantly influenced by boar breed: significant differences were also found within some signs for the body parts examined. Figures 5 shows a comparison of offspring from Duroc boars with those of the Pietrain boars with the lowest (PI-L: boar number 19) and the highest SINS scores (PI-H: boar number 8) and with the average of all Pietrain offspring (PI-A). Boar (*p* = 0.002) and breed (*p* < 0.001) had a significant influence on the clinical outcome of inflammation and necrosis in the tail base (Fig. 5a). The prevalence of piglets without bristles, with swelling, exudation and necrosis was significantly affected by the boar; loss of bristles and swelling were additionally affected by the breed. The proportion of piglets with alterations in the tail base was 75% lower in the Duroc offspring than in the Pietrain offspring. Within Pietrain, twice as many piglets from boar 8 (highest SINS score) showed alterations in the tail base than piglets from boar 19 (lowest SINS score). Only the redness exhibited tendential difference. Necrosis at the tail base occurred only in some offspring of Pietrain boars.

In contrast to the base of the tail, the remaining tail (tail tip) was significantly less affected in the suckling piglets examined (Fig. 5b). A maximum of 28.8% of the piglets were affected by a single alteration. Significant effects of boars were found only for swelling and exudation of the tail (*p* < 0.05). The breed had no significant effect, but bleeding and necrosis occurred only in some piglets from Pietrain boars. At the ears of the suckling piglets examined, the loss of bristles and congested ear veins were particularly noticeable (Fig. 5c). Only the influence of the breed on the loss of bristles was statistically significant. Ear necrosis occurred only sporadically and only in offspring of the Pietrain boars. Oedema at the eyelids and the nose were significantly more prevalent in Pietrain offspring than in Duroc offspring (Fig. 5d). Teats were significantly affected by boar (*p* < 0.05) and breed (*p* < 0.05). While only marginal changes in teats were observed in the offspring of Duroc boars, such changes occurred at a high frequency in Pietrain offspring, particularly in the form of swelling and venous congestions (Fig. 5e). The offspring of extreme boars within the Pietrain breed (PI-L: boar 19 and PI-H: boar 8) exhibited statistically significant differences (*p* < 0.05). The claws were also significantly influenced by the boar breed. However, there were hardly any significant differences between boars within breeds, with the exception of heel swelling and heel redness and the bulging of the claw wall. Nevertheless, overall changes in the claws were twice as frequent in Pietrain offspring compared to Duroc offspring. The offspring of the most favourable Pietrain boar were affected a quarter less frequently than the offspring of the least favourable Pietrain boar (Fig. 5f). No significant effects around the navel were found (data not shown) in the present study.

**Fig. 6:**
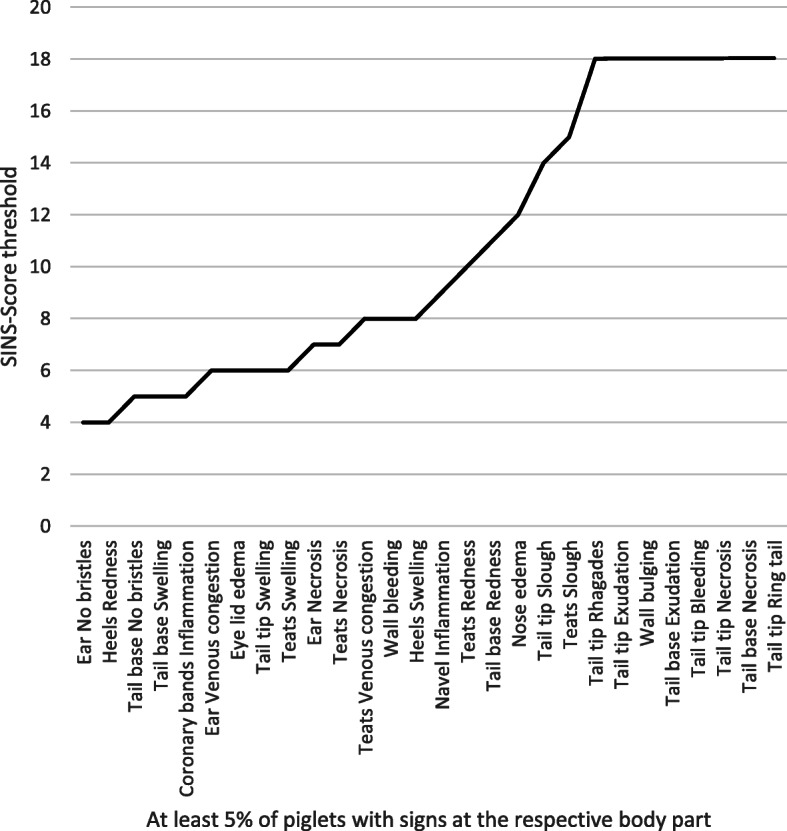
SINS score threshold for signs on the examined body parts. Shown is the SINS score above which at least 5% of the piglets were affected. With SINS scores from 18 on, less than 5% of the piglets in total were affected by the corresponding alterations on the respective body part

The first signs of SINS (signs that were already present at lowest SINS scores) were loss of bristles on the ears and at the base of the tail, redness on the heels, coronary band inflammation and swelling of the base of the tail (Fig. 6). This figure shows the threshold for the SINS score where at least 5% of the piglets were found with the respective sign. Signs like rhagades, exudation, bleeding and necrosis of the tail, as well as exudation and necrosis of the tail base and wall bulging were found in less than 5% of the piglets, even in those with the highest SINS scores.

## Discussion

The study shows that the prevalence of inflammation and necrosis at different body parts was affected by boar breed and by the individual boar in two consistent sow herds. The findings correspond well with findings previously described in piglets [14, 16, 17]. Forty percent of the piglets showed signs in at least 5 of 7 body parts (where heels, coronary bands and claw walls were grouped together as claws). Loss of bristles, swelling and redness were most common in this age group, but sloughs, exudation and necrosis were also visible at different body parts in one to 10 % of the piglets.

For the purpose of a better applicability under field conditions, SINS diagnostics has been based in particular on visually detectable clinical signs. This also enables the farmer to recognise and react as early as possible. The signs were presented in detail in two studies using photographs of the different SINS-stages [14, 15]. The association of the visual clinical signs with the underlying histopathology was also proven [16, 17]. Even in animals with macroscopically intact epidermis, considerable prevalence of vasculitis, intima proliferation and thrombosis of the vessels were detected [17], including newborn piglets that had no time to develop signs solely from biting or technopathy [16].

For the evaluation of the SINS signs, numerous body parts (base of the tail, tip of the tail, ears, face, teats, navel, coronary bands, claw walls and heels) were included and evaluated qualitatively mainly for the occurrence of bristle loss, redness, swelling, exudation and necrosis. The individual findings are presented in detail in Reiner et al. [14] and Reiner and Lechner [15]. This binary system appears much easier and more comparable to use than further quantification of the individual clinical sign. Since in the present study all data were collected synchronously from two persons, errors due to inter-observer effects can be excluded. However, such effects were also not the explicit subject of the study.

According to EFSA [1, 2], inflammation and necroses can seriously impair animal welfare. Injuries to the skin, tail and ears are generally used as valuable characteristics for assessing animal welfare in pigs [20–22]. Therefore, the findings presented here are likely to be relevant diagnostic indicators of impaired animal welfare in swine. The observed degrees from bristle loss to necrosis vary considerably. However, histopathological studies show that bristle loss (at least proven for the tail base) is associated with the accumulation of inflammatory cells and inflammatory alterations in the area of the bristle papillae [16]. Therefore, the loss of bristles is at least of importance for the early detection of SINS. Whether and to what extent the accompanying inflammation itself already leads to pain and suffering in the affected animals cannot yet be answered.

Lesions to ears, tail and skin are generally highly prevalent in pig production systems [23]. Previous studies have shown that the degree of inflammation and necrosis (SINS) is influenced by husbandry and the quality of the sow [17]. Associations between ear, tail and skin lesions and production conditions have also been described by other authors [23]. Alopecia, swelling, coronary band injuries and swelling and haemorrhaging into the solar corium have also been described in suckling piglets by Mouttotou et al. [24] and KilBride et al. [25]. The authors suppose that some of these lesions are associated with a reduction in suckling and active behaviour and a slower growth rate because of the pain associated with such injuries [26]. The authors attributed the changes solely to mechanical irritation by the floor. Recently, clear evidence for an internal component in such lesions was provided by histopathological findings [17].

Effects of sow genetics on the prevalence and severity of SINS in suckling piglets has also been described [14]. A comparison between Duroc and Pietrain offspring from two uniform sow herds in this study showed a completely separate distribution. The SINS confidence intervals for Duroc offspring (4.5–5.2) were significantly lower than for Pietrain offspring (7.9–8.4) and indicate a largely complete separation of the breeds, at least based on the boars used for the study. This results in a significantly more favourable prevalence of inflammation and necrosis in the various organs of their offspring when Duroc boars are used under the same husbandry and feeding conditions and with the same sow line.

The use of Duroc boars instead of Pietrain boars resulted in a 59% reduction in the SINS scores of their offspring in the present study. For the individual organ systems of the tail base, tail tip, ears, face, teats, navel and claws, this resulted in a reduced inflammation/necrosis prevalence of 74, − 20%, 24, 46, 88, 63 and 52%, respectively. The increased alterations in the area of the tail tip were low, because such changes were rare compared to changes in the tail base in the investigated age class [17]. The current experiment also showed significant differences when comparing the offspring of different Pietrain boars. The offspring of the most favourable Pietrain boar had a 37% lower SINS score than the least favourable, while the inflammation/necrosis prevalence for tail base, tail tip, ears, face, teats, navel and claw was reduced by 54, 53, 15, 17, 61, 82 and 26%, respectively.

This study was triggered by findings from practical experience, revealing offspring of different boars in the same herd to develop different levels of SINS signs. Such findings are currently being taken seriously by numerous breeding companies in various countries. As there is no scientific data available on this issue, boars of different breeds and breeding companies were selected on the basis of such practical observations and examined according to the sensitivity of their offspring to SINS under constant environmental conditions. The boars were specifically selected so as to exhibit the widest possible range of SINS-outcome. Therefore, the results do not allow a representative overview of the participating breeding companies or their lines. An exact disclosure of the boars used is therefore neither possible nor meaningful for scientific reasons. The results clearly show that in individual breeding companies there are boars with both favourable and unfavourable distribution of SINS in their offspring. However, the decisive results of the study are the clear differentiation between Duroc and Pietrain boars and the pronounced variation within the Pietrain boars. It would be desirable to include a wider range of boars in future work and to investigate correlates between SINS sensitivity in the offspring and the different aspects of boar performance.

Since it can be assumed that the expression of the SINS signs is significantly influenced by husbandry and feeding [14, 17], it can be suspected that the absolute differences between boars should be weaker or stronger under more favourable or less favourable conditions. In the present study, however, the initial aim was to determine boar differences based on their offspring under constant environmental conditions. The experiment was not designed to detect effects of individual husbandry and feeding conditions on the expression of SINS, but on the differences between boars. Follow-up studies are necessary, e.g. to study the offspring of two extreme boars on farms with defined differences in husbandry and feeding.

SINS studies prove by histopathological examination both the inflammatory basis of the disease [17] and the development of signs in newborns who have not been exposed to bites or technopathies [16], which, together with the inclusion of different body parts in the syndrome such as tail, ear, claws and teats, indicates a primarily endogenous aetiology. Circulatory disorders in the tail area of suckling piglets, which can lead to necroses, have already been suspected in earlier studies [11–13]. The histopathological evidence of vasculitis and thrombosis in the area of the tail base proximal to the lesions in affected piglets [16, 17] confirms this suspicion. A circulatory disorder was also clinically proven by an abrupt drop in temperature within this part of the tail base of affected piglets measured by thermal imaging camera [15]. Our hypothesis for the explanation of SINS is therefore based on local inflammatory processes in the vicinity of the blood vessels [15]. Various authors showed associations between deoxynivalenone (DON) and lipopolysaccharides (LPS) from the sow and necroses on the tail, ears or coronary bands of piglets [27–29]. LPS is one of the strongest activators of the macrophages and cellular defence mechanisms [30], but it is just one example of numerous bacterial degradation products that activate the defence and immune system and trigger inflammation in the sense of PAMPs [31]. The effects of inflammation, e.g. on the basis of LPS, are likely to be not only limited to local reactions, but also lead to massive reactions in the CNS that can cause sickness behaviour, Post Partum Dysgalactia Syndrome (PPDS [32, 33]), and that can even trigger tail biting [34, 35]. Nordgreen et al. [35] give a comprehensive synthesis of how problems in the areas of microbiota, blood-gut barrier, housing environment and hygiene, immune activation, mycotoxins, psychological stress, nutritional status and feed composition can synergistically lead to inflammation directly or indirectly in association with LPS, and how the CNS is influenced by the inflammation mediators. Here our hypothesis of the development of SINS [15] as a further local component in addition to the central effects fits perfectly in.

An important source for the flooding of LPS is the intestine (for discussion see Nordgreen et al. [35], especially under high protein/crude fibre ratios, intestinal diseases and disturbances to the blood-gut barrier [36]. The blood-gut barrier in pigs is particularly susceptible to heat stress [37–40], reduced intestinal perfusion [38] and mycotoxins [41, 42]. Mycotoxins and LPS can interact synergistically, potentiating each other’s uptake from the gut and disturbing each other’s degradation in the liver [41–45]. Furthermore, psychological stress e.g. because of missing environmental enrichment does appear to cause inflammatory reactions, which are similar to those seen as a response to LPS challenge [35]. And all these factors, thermoregulation, water supply (quantity and quality), high protein and starch content in the feed, low crude fibre content, mycotoxin contamination and stress are commonly highlighted in the context of husbandry problems in pig production, often together with tail biting and tail lesions (e.g. [4, 6]). They are also candidates for the main determining factors in SINS.

On the basis of this hypothesis, a number of candidate genes with involvement in e.g. liver metabolism, inflammatory metabolism and the blood-gut barrier could be defined, which in the case of functional gene variants should lead to different degrees of SINS in affected piglets under otherwise identical environmental conditions, as shown in the current study. One example of such a candidate gene would be SPONDIN-1, a gene described as promoting the release of cytokines such as interleukin 6 as an inflammatory response, e.g. to LPS [46]. Variants of this gene have recently been described with association to the sensitivity of pigs to LPS after infection with *Actinobacillus pleuropneumonia* [47]. However, elucidating the pathways and pathomechanisms involved, and thereby identifying the reasons for the different sensitivities to SINS of offspring of different boars still requires a substantial amount of research.

## Conclusion

The results suggest that selecting suitable boars, even within the Pietrain breed, can significantly reduce inflammation and necrosis in their offspring. In addition to improving husbandry and feeding, genetic selection should also be used to combat inflammation and necrosis in suckling piglets. However, no data are yet available on the heritability of SINS traits or on breeding values of boars. In view of the great importance of inflammation and necrosis for animal welfare in pigs, such data should definitely be collected.

## Data Availability

The datasets used and analysed during the current study are available from the corresponding author on reasonable request.

## Abbreviations

SINS: Swine Inflammation and Necrosis Syndrome
